# The effect of prestimulus low-frequency neural oscillations on the temporal perception of audiovisual speech

**DOI:** 10.3389/fnins.2023.1067632

**Published:** 2023-02-03

**Authors:** Zeliang Jiang, Xingwei An, Shuang Liu, Lu Wang, Erwei Yin, Ye Yan, Dong Ming

**Affiliations:** ^1^Academy of Medical Engineering and Translational Medicine, Tianjin University, Tianjin, China; ^2^Defense Innovation Institute, Academy of Military Sciences (AMS), Beijing, China; ^3^Tianjin Artificial Intelligence Innovation Center (TAIIC), Tianjin, China

**Keywords:** EEG, neural oscillations, speech, audiovisual, temporal integration

## Abstract

**Objective:**

Perceptual integration and segregation are modulated by the phase of ongoing neural oscillation whose frequency period is broader than the size of the temporal binding window (TBW). Studies have shown that the abstract beep-flash stimuli with about 100 ms TBW were modulated by the alpha band phase. Therefore, we hypothesize that the temporal perception of speech with about hundreds of milliseconds of TBW might be affected by the delta-theta phase.

**Methods:**

Thus, we conducted a speech-stimuli-based audiovisual simultaneity judgment (SJ) experiment. Twenty human participants (12 females) attended this study, recording 62 channels of EEG.

**Results:**

Behavioral results showed that the visual leading TBWs are broader than the auditory leading ones [273.37 ± 24.24 ms vs. 198.05 ± 19.28 ms, (mean ± sem)]. We used Phase Opposition Sum (POS) to quantify the differences in mean phase angles and phase concentrations between synchronous and asynchronous responses. The POS results indicated that the delta-theta phase was significantly different between synchronous and asynchronous responses in the A50V condition (50% synchronous responses in auditory leading SOA). However, in the V50A condition (50% synchronous responses in visual leading SOA), we only found the delta band effect. In the two conditions, we did not find a consistency of phases over subjects for both perceptual responses by the *post hoc* Rayleigh test (all *ps* > 0.05). The Rayleigh test results suggested that the phase might not reflect the neuronal excitability which assumed that the phases within a perceptual response across subjects concentrated on the same angle but were not uniformly distributed. But *V*-test showed the phase difference between synchronous and asynchronous responses across subjects had a significant phase opposition (all *ps* < 0.05) which is compatible with the POS result.

**Conclusion:**

These results indicate that the speech temporal perception depends on the alignment of stimulus onset with an optimal phase of the neural oscillation whose frequency period might be broader than the size of TBW. The role of the oscillatory phase might be encoding the temporal information which varies across subjects rather than neuronal excitability. Given the enriched temporal structures of spoken language stimuli, the conclusion that phase encodes temporal information is plausible and valuable for future research.

## 1. Introduction

Audiovisual speech plays an essential role in our lives. The integration of visual mouth movements and auditory voice can promote spoken language recognition (Crosse et al., 2015), even in noisy environments (Zion Golumbic et al., 2013). The temporal interval between stimuli is essential for integration. If the interval of two stimuli falls within a specific time window, which is often referred to as Temporal Binding Window (TBW) (Vroomen and Keetels, 2010), the two stimuli are more likely to be integrated. More recently, the phase of ongoing neural oscillations, especially in lower frequency bands (< 20 Hz), has been proven to be related to the process of perceptual integration or segregation in the visual (Ronconi et al., 2017) and somatosensory (Baumgarten et al., 2015) domains. In addition, using simultaneity judgments (SJ) tasks and low-level beep-flash stimuli, it has been indicated that synchronous or asynchronous responses to audiovisual stimuli were highly related to the phase of alpha-band oscillation activities (Ikumi et al., 2019). However, the phase effect of spontaneous neural oscillations on audiovisual speech temporal perception remains unclear.

Previous research shows that the frequency of spontaneous neural oscillations associated with perceptual integration or segregation changes as a function of the TBW size in the visual domain (Ronconi et al., 2017). More specifically, the oscillation periods of brain neurons should match with or be larger than the duration of TBWs. For example, if the TBW size is about 80 ms, the corresponding oscillation frequency should be with a period larger than 80 ms, that is, any oscillation under 12.5 Hz (ten Oever and Sack, 2015). The behavioral studies about beep-flash stimuli have indicated that the TBW size is about 100 ms (Zampini et al., 2005; Stevenson and Wallace, 2013). An EEG study further found that the TBW for beep-flash stimuli is about 80 ms modulated by the alpha band (13 ± 2 Hz) phase (Ikumi et al., 2019). In addition, a large number of studies have shown that the phase of the delta and theta frequency bands (2–7 Hz) is related to spoken audiovisual stream tracking (Luo et al., 2010), disambiguation (Thézé et al., 2020), and syllable recognition (ten Oever and Sack, 2015). Moreover, the TBW increases to a few hundred milliseconds for more complex audiovisual speech stimuli (Stevenson and Wallace, 2013; De Niear et al., 2018). Based on the size of TBW and the frequency response associated with spoken stimuli, we speculated that cross-modal synchrony perception for speech could be modulated by the phase of delta and theta frequency bands whose periods are ranging from 150 to 1,000 ms (1–7 Hz).

We also consider two kinds of audiovisual stimuli conditions: auditory-leading condition (auditory stimuli were onset before visual stimuli) and visual-leading condition (visual stimuli were onset before auditory stimuli). Previous studies have revealed that the size of TBW for speech stimuli is asymmetric: visual-leading TBW (Right TBW; RTBW: about 250 ms) is significantly larger than auditory-leading ones (Left TBW; LTBW: about 150 ms) (Stevenson and Wallace, 2013). This phenomenon can be explained by Schwartz and Savariaux’s study which found that audiovisual asynchronies for the natural audiovisual speech materials vary between 20 ms audio lead and 70 ms audio lag (Schwartz and Savariaux, 2014). Moreover, it has been indicated that the alpha band phase modulated cross-modal synchrony perception in the auditory leading condition for beep-flash stimulus (Ikumi et al., 2019). So we supposed that for the speech stimuli the RTBW might be modulated by the 7 Hz oscillation within the theta band (4–7 Hz) while the LTBW might be modulated by the 4 Hz oscillation within the delta band.

Besides, there might be different oscillation rhythms biasing perceptions within the same TBW. Theoretically, when two stimuli fall within the same cycle of a neural oscillation, they tend to be integrated (Varela et al., 1981). That is, as long as the width of the TBW is smaller than the period of a certain neural oscillation, the neural oscillation of that frequency might modulate whether the two stimuli are integrated or not. People could recognize the spoken language of the other speakers with varying speaking rates and could easily switch among them. Moreover, researchers have suggested that different entraining frequencies shape the perception of the same speech stimulus (ten Oever and Sack, 2015; Kösem et al., 2018). Furthermore, many studies show that TBW can be narrowed by perceptual training (De Niear et al., 2018; McGovern et al., 2022). The main reason is that our brain can freely learn the onset differences of audiovisual modality for speech and use multiple rhythms to parse different temporal structures. So we assumed that the LTBW could be also modulated by the delta band phase whose period is larger than 250 ms, while the RTBW could be modulated only by the delta band phase.

Last, if the phase can modulate the synchronous perception of audiovisual speech, we also want to explore its functional role. At present, there are two views to explain the functional role of phase. Most of the non-speech papers suggest that the phase is related to the cyclic shifts in neuronal excitability (Busch et al., 2009; Milton and Pleydell-Pearce, 2016; Ikumi et al., 2019). For instance, Milton and Pleydell-Pearce found that the mean alpha phase angle across participants is near-opposite between two perceptual responses: it is about 270° for asynchronous responses which reflect raised excitability while 90° for simultaneous responses which reflect decreased excitability (Milton and Pleydell-Pearce, 2016). However, the speech-related paper argued that phase plays a role in coding temporal information (ten Oever and Sack, 2015; Zoefel et al., 2018). For example, a study using an ambiguous syllable recognition task in which the audiovisual onset difference of syllables is different found that the phases at which the same syllable was recognized varied across participants (ten Oever and Sack, 2015). So we speculated that the role of the phase may be different for speech and non-speech stimuli.

All in all, in this paper, we will conduct the simultaneity judgment (SJ) tasks paradigm to explore if the oscillation phases modulate the temporal perception of the speech stimulus. If any, what is the functional role? The chapter arrangement of this paper is organized as follows. In see Section “2. Materials and methods,” we describe the experimental materials and methods including participants, stimuli, experimental design, and data analysis. See Section “3. Results” gives a systematic description of the experimental results including behavioral results, as well as the EEG results. See Section “4. Discussion” discusses the results. See Section “5. Conclusion” presents the conclusions of this paper.

## 2. Materials and methods

### 2.1. Participants

In this study, 20 participants (8 males; 18–29 years old) volunteered to participate in the experiment. One subject was left-handed, and the others were all right-handed subjects. The sample size was determined based on previous literature and G-power software. Specifically, previous EEG studies on the role of the pre-stimulus phase on temporal perception considered a sample size of between 8 and 27 participants (Milton and Pleydell-Pearce, 2016; Ronconi et al., 2017; Ikumi et al., 2019; Bastiaansen et al., 2020). In addition, we use G-power 3.1 software (Faul et al., 2007) to determine the minimum sample size required to test the study hypothesis. Results indicated the required sample size to achieve 80% power for detecting a large effect, at a significance criterion of α = 0.05, was *N* = 15 for one-sample and paired-sample *t*-tests. Thus, the obtained sample size of *N* = 20 is more than adequate to test the study hypothesis. They all had normal or corrected to normal vision and normal hearing. All recruitment and experimental procedures were approved by the Tianjin University Institutional Review Board.

### 2.2. Stimuli

**FIGURE 1:**
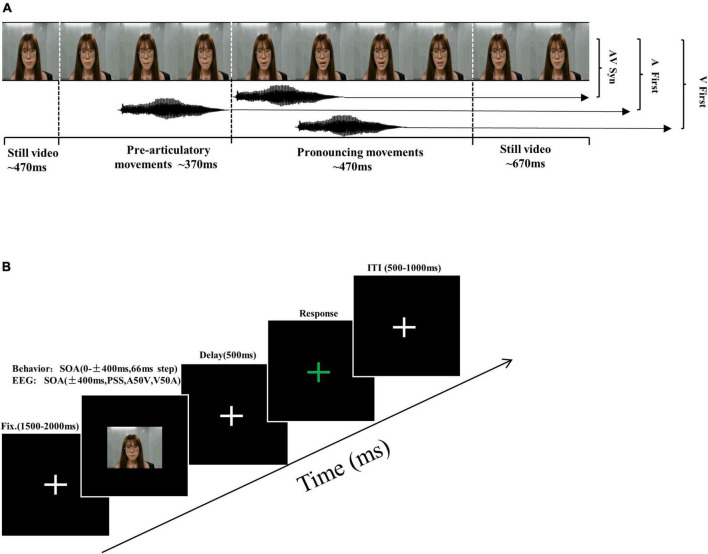
Schematic of trial types and experimental procedure. **(A)** Different stimulus onset asynchronous (SOA) stimuli conditions were created by adjusting the relative onset time of the acoustic tracks and the visual frames. The SOA in which the acoustic signal is presented before the mouth just opened was defined as auditory leading SOA. The SOA in which the acoustic signal is presented after the mouth just opened was defined as visual leading SOA. The original movie in which the acoustic signal is presented at the same time with the mouth just opened was defined as 0 ms SOA. **(B)** Overview of structure and timing of a single trial. Participants provided non-speeded, two-alternative forced-choice (2AFC) simultaneity judgments in the response interval.

The experimental stimuli consisted of videos of a female speaker uttering the syllable/ba/slower than normal speed, similar to numerous prior studies (De Niear et al., 2018; Simon and Wallace, 2018), with a resolution of 720 × 480, 29.97 Hz Frame Rates, and a duration of 2 s. The original video consisted of 470 ms of rest, 370 ms of pre-articulatory movement, 470 ms of pronouncing action, and 670 ms of rest after the end of the movements (see Figure 1A). To create different audiovisual stimulus onset asynchronous (SOA) conditions, we use Adobe Premiere Pro 2020 software to adjust the time difference between the acoustic track and the mouth movement. The SOA in which the acoustic signal is presented before the mouth just opened was defined as auditory leading SOA. The SOA in which the acoustic signal is presented after the mouth just opened was defined as visual leading SOA. The original movie in which the acoustic signal is presented at the same time with the mouth just opened was defined as 0 ms SOA (Simon and Wallace, 2018). Of note, because we defined the mouth just opening as the moment of the visual key stimulus onset in the visual leading SOA or the acoustic signal onset as the moment of the auditory key stimulus onset in the auditory leading SOA, the pre-articulatory movement was always before the key stimulus onset.

### 2.3. Experimental design

#### 2.3.1. Pre-test behavior session

The experiment comprised a pre-test behavior session and one EEG session based on a simultaneity judgment (SJ) task (Figure 1B). Pre-test behavior session aimed at estimating the audiovisual asynchronies threshold of maximal perceptual uncertainty (∼50% responses of each category), which were called A50V (50% synchronous responses in auditory leading SOA) and V50A (50% synchronous responses in visual leading SOA). In the following EEG sessions, the two thresholds were used to ensure a relatively balanced ratio of synchronous and asynchronous responses.

In this task, we defined 13 different SOAs: ± 400, ± 333, ± 266, ± 200, ± 133, ± 66, and 0 ms. Negative SOAs indicated the auditory signals were first presented. The videos were presented on a 23-in monitor (Philips 236V6Q) with a refresh rate of 60 Hz at a distance of 0.6 m in front of the subjects. Auditory signals were played through an In-Ear-Monitor (EDIFIER H230P). The sounds were adjusted to a comfortable hearing level. All speech stimuli were presented using E-Prime version 3.0.

In the pre-test experiment, the method of constant stimuli was used to estimate the individual thresholds. There were 20 trials for each SOA, divided into four blocks. The experimental process was as follows: First, a white central cross appeared on the screen lasting about 1,500–2,000 ms, followed by a video stimulus lasting 2,000 ms. Second, the white central cross emerged again for 500 ms as a delay interface to prevent the subjects’ motor preparation signal from interfering with the EEG signals. Then, a response interface appeared. At this point, the white cross turned green. The subjects were asked to perform the two-alternative forced-choice (2AFC) simultaneity judgment task to determine whether the visual mouth movements and acoustic sounds were synchronous. Press the button “F” when the video was synchronous, and press the button “J” when it was asynchronous using the keyboard. The interface would not disappear until the participants made a judgment. Finally, an inter-trial interval (ITI) appeared, lasting 500–1,000 ms. In the whole process, different conditions appeared randomly (see Figure 1B).

#### 2.3.2. Thresholds and window size estimation

**FIGURE 2:**
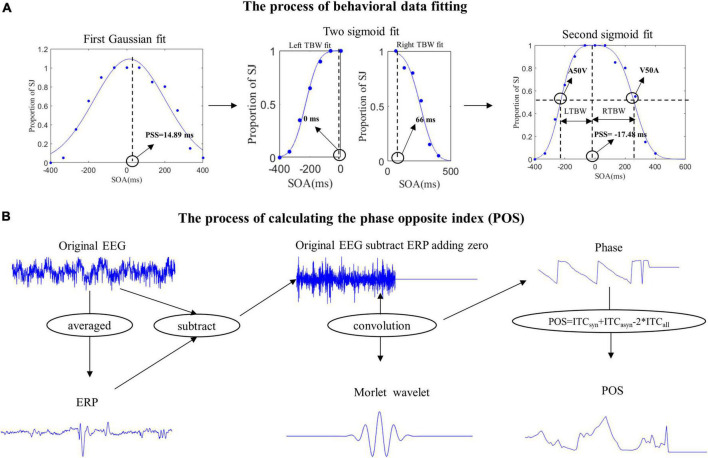
The pipelines of behavior data fitting and calculating phase opposite index (POS). **(A)** We first fitted the Gaussian curve to get the point of subjective simultaneity (PSS). Then two sigmoid curves were fitted with the stimulus onset asynchronous (SOAs) located to the left and right of the PSS to get the new PSS, A50V, and V50A thresholds. The left temporal binding window (LTBW) was defined as the difference between PSS and A50V. The right temporal binding window (RTBW) was defined as the difference between PSS and V50A. **(B)** In our experiment, evoked activities were induced from the white central cross to the onset of the video and from the visual face onset to the visual preparatory movement onset. To exclude the effect of evoked activity on the ongoing induced activity, we first computed the ERP (the time-domain trial average) and then subtracted the ERP from the original EEG signal on each trial. This was done separately for each SOA condition, electrode, and subject. Then we padded all data points after zero the amplitude value of zero μV to exclude the poststimulus effects temporally smearing back to the prestimulus interval. Furthermore, we used Morlet wavelets to extract the phase of ongoing EEG oscillations and quantified the phase opposite between synchronous and asynchronous response with the POS.

According to the curve fitting methods used by De Niear et al. (2018), we use two steps to get the A50V or V50A thresholds and window size (De Niear et al., 2018). The proportions of synchronous responses in the pre-test behavior session were first fitted individually with Best-fitting Gaussian curves (least-squares minimization using iterative maximum likelihood) by Matlab cftool toolbox. The PSS (Point of Subjective Simultaneity, the point with the highest proportion of synchronous responses) was derived as the peak of the SJ Gaussian probability function. If the PSS is 14.89 ms, the proportions of synchronous responses at each SOA which is greater than 66 ms would be fitted with an individual sigmoid curve while the data at each SOA which is smaller than 0 ms would be fitted with another sigmoid curve by Matlab glmfit function. The A50V or V50A was the point of the 50% proportion of synchronous responses in audition-leading or vision-leading fitting curves. The new PSS was defined as the intersection of the two fitting sigmoid curves (Figure 2A). The difference between the A50V or V50A and PSS was defined as the width of the left or right (audition or vision presented first) temporal binding windows. The goodness of fit (*R*^2^) was 93.10 ± 6.4% for left and 84.00 ± 19.67% for right curve fitting respectively on average across participants (mean ± SD). One subject’s data was removed due to poor fitting results (*R*^2^ is 93.66 and 3.45% for the left and right curve fitting respectively).

#### 2.3.3. EEG session

In the EEG session, similar experimental setups for each trial were conducted with five different SOAs (± 400 ms, PSS, A50V, V50A). We used the A50V and V50A derived from the pre-test behavior session as the main stimuli condition. PSS and ± 400 ms SOAs as filling stimuli were added to prevent subjects from responding randomly. Each participant completed eight blocks of 70 trials. The A50V and V50A conditions were each repeated 26 times while the PSS and ± 400 ms SOAs were each repeated six times in each block (Yuan et al., 2016).

#### 2.3.6. EEG acquisition and preprocessing

A SynAmps amplifier system (Neuroscan) with 62 Ag/AgCl electrodes was placed according to the international 10–20 system with a sampling rate of 1,000 Hz for EEG recording. Electrode impedances of each electrode were kept below 5 KΩ. The filter bandpass was 0.01–200 Hz. Signals were referenced online against the left mastoid. All subsequent data processing was undertaken in Matlab (MathWorks) using EEGLAB Toolbox (Delorme and Makeig, 2004), Fieldtrip Toolbox (Oostenveld et al., 2011), and Circular Statistics Toolbox (Berens, 2009).

All the preprocessing steps of EEG data were completed using the EEGLAB toolbox. EEG channels were firstly re-referenced to two linked electrodes on the left and right mastoid and down-sampled to 500 Hz. Then the data were band-pass filtered (1–80 Hz) and notch filtered (around 50 Hz) with a Hamming windowed-sinc finite impulse response zero-phase filter. The ICA algorithm and ADJUST plugin of EEGLAB were used to remove the artifacts of horizontal and vertical electrooculogram, ECG, and EMG. The clear data were first segmented into long epochs defined as −4,000 to 4,000 ms post-onset of the video. Since the markers were located at the time of video onset, we needed to adjust the marker points to the time of each subject’s specific auditory or visual signal onset. Data were epoched −3 to 2.5 s around syllable onset finally. Epochs containing voltage deviations exceeding ± 100 μV were excluded.

### 2.4. EEG analysis

#### 2.4.1. Spontaneous neural oscillation analysis

Although there is an ongoing debate about the relationship between ongoing and evoked activity, evoked activity is often considered to be independent of ongoing brain activity (Scheeringa et al., 2011). Many studies assumed that the ongoing activity and evoked activity were additive (Qian and Di, 2011; Cohen and Donner, 2013). They tend to subtract the ERP from the original time-domain EEG signal on each trial to get ongoing-induced oscillatory activity (Cohen and Donner, 2013; Beatty et al., 2020).

In our experiment, evoked activities could be induced from the white central cross to the onset of the video and from the visual face onset to the visual preparatory movement onset. So the baseline defined in our research was contaminated by the evoked activity. To get the ongoing activity, just like Keil et al. (2012), we performed two steps to remove the effects of evoked activity in the following way (Keil et al., 2012). Given that the stimulus was the same between the synchronous and asynchronous response before the key stimulus onset, we first averaged over all trials across the different responses separately for each SOA condition, electrode, and subject to get the ERP. Then we subtracted the ERP from the original EEG signal on each trial to get the ongoing neural oscillations.

After obtaining the ongoing neural oscillatory activity, we furthermore padded all data points after zero ms with the amplitude value of zero μV to exclude the poststimulus effects temporally smearing back to the prestimulus interval which has been used by other related studies (ten Oever and Sack, 2015; Grabot and Kayser, 2020). To extract the phase of the ongoing EEG oscillations, the complex Fourier spectrum of the single trial was then computed using the Morlet wavelets transform between 1 and 13 Hz (The number of cycles used linearly increasing from 1.5 at 1 Hz to 9.75 at 13 Hz) with a step size of 0.5 Hz between −3 and 2.5 s around the first stimulus onset. Because the wavelet transform will spread the pre-stimulus data to the post-stimulus, to make full use of the pre-stimulus data, we extend our time range of interest from 800 ms before the stimulus onset to 200 ms after the stimulus onset.

Then we use Phase Opposition Sum (POS = ITC_syn_ + ITC_asyn_–2ITC_all_; ITC_syn_ and ITC_asyn_ are the mean resultant vectors of single-trial phase angles for trials where a synchronous or asynchronous response was perceived, respectively. ITC_all_ is the mean resultant vector of single-trial phase angles for all trials together, irrespective of perception) to evaluate whether the prestimulus phases were concentrated around a mean value statistically different between synchronous and asynchronous responses (VanRullen, 2016). Theoretically, the POS values vary between 0 and 2. The POS value is 0 when phase distributions for each response are fully random or when both responses have the same phase angle. A value of 2 reflects a perfect phase opposition between the two responses. Intermediate POS values represent a partial phase opposition where the response choices were modulated by near-opposite phases within subjects.

Intertrial phase coherence (ITC) is easily affected by the number of trials (Hanslmayr et al., 2013), and different responses have an unequaled number of trials in our experiment. Thus to ensure that effects were not due to differences in trial numbers, we performed trial selection employing random sub-sampling 100 times of the higher number of trials to the lower number of trials and subsequently computed the mean of the POS over these 100 repetitions as an empirical level POS. For statistic analysis, we recalculated the POS by randomly shuffling the labels of the two responses and randomly selecting the same number of trials which is equal to the trial number of the response with the lowest trial number for the two surrogate groups. We repeated this procedure 100 times and took the average of this as our final chance level POS (Wolpert and Tallon-Baudry, 2021). Figure 2B shows the whole flow of calculating POS.

### 2.5. Statistical analysis

#### 2.5.1. Behavior data statistical analysis

A single sample *t*-test tested the proportions of synchronous responses under the condition of A50V and V50A to verify whether the averaged ratios of synchronous responses were around 50%. Then the behavior data under the five SOAs were measured by repeated one-way ANOVA analysis. When the spherical test was violated, the Greenhouse-Geisser method was used for correction, and the Bonferroni method was used for *post hoc* multiple comparisons.

#### 2.5.2. EEG statistical analysis

A cluster-based (at least two sensors per cluster) dependent-samples *t*-test with Monte-Carlo randomization 1,000 times was performed to find the difference of POS (right-tailed) between the empirical level POS and the chance level POS. The test is one-tailed because the hypothesis is that there is more phase coupling than expected by chance. This method allowed for the identification of clusters of significant differences in 2D (time and space) and 3D (time, frequency, and space) data, and effectively controlled multiple comparisons (Maris and Oostenveld, 2007). The process of cluster-based permutation test is as follows: A paired-sample *t*-test is performed first across time, frequency, and sensors. Then all *t* values above a threshold (uncorrected *p* < 0.05, right tail) were connected into clusters by grouping adjacent significant time-frequency and electrode points (at least 6 adjacent data points). Next, we summed the *t* values within each cluster to get a cluster-level *t* value (cluster statistic). Subsequently, the two response labels were permutated 1,000 times to get the null distribution of cluster-level *t* value by repeating the above process based on the Monte Carlo randomization method. Finally, if a given cluster had a *t* value higher than 95% of the null distribution, then this was considered a significant effect (5% alpha level, right tail).

#### 2.5.3. Phase angles and perception

Since the POS is an indirect index to quantify the phase opposite between the synchronous and asynchronous response across subjects. To characterize the direct phase effect among subjects and test the functional role of the phases, we extracted and computed for each subject the average phase angles for both perceptual responses across the time-frequency and electrode points showing the largest statistical phase angle effect (maximum *t* value of the cluster in the aforementioned POS statistics) for further statistical tests. The logic behind the test of the functional role of the phases is that if the phases represented cyclic shifts in neuronal excitability, the phases across subjects will concentrate on the same phase for the synchronous or asynchronous response, and the phase between the synchronous and asynchronous response will be opposite within subjects, the phase difference between two perceptual responses will concentrate around π across subjects. However, if the phases coded temporal information of audiovisual modality onset differences, the phases across subjects might not concentrate on the same phase for the synchronous or asynchronous response, and the phase difference between the synchronous and asynchronous response will also concentrate around π. First, we used the Rayleigh test to investigate if the mean phase over participants was consistent for both perceptual responses respectively (Berens, 2009). Then we performed the *V*-tests to explore whether the phase difference between the synchronous and asynchronous responses is non-uniform and around π (ten Oever and Sack, 2015; Ten Oever et al., 2020).

## 3. Results

### 3.1. Behavior results

**FIGURE 3:**
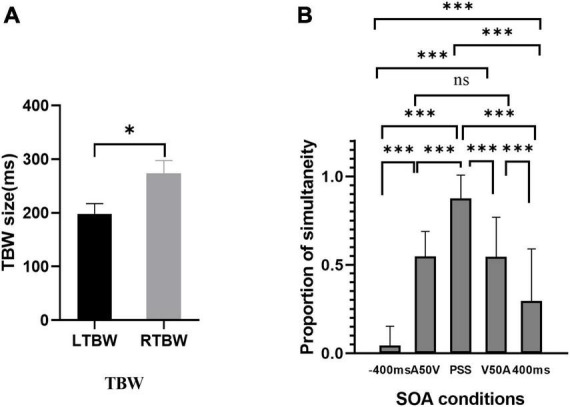
The results of behavior responses in two experimental sessions. **(A)** Auditory and visual leading temporal binding windows across subjects are significantly different. **(B)** Behavioral results of the EEG session. The proportion of synchronous response is shown for each stimulus onset asynchronous (SOA). *p < 0.05, ^***^p < 0.001. ns, not significant.

The results of paired sample *t*-test in the pretest behavior session showed that the width of TBWs in visual leading SOAs was larger than those in auditory leading ones [*t*_(18)_ = 2.46, *P* = 0.024, Cohen’s *d* = 0.564; TBW_*A*50*V*_ = 198.05 ± 19.28 ms, TBW_*V*50*A*_ = 273.37 ± 24.24 ms (MEAN ± SEM), Figure 3A]. The proportions of synchronous responses in EEG sessions (Figure 3B) were submitted to repeated-measures one-way ANOVA with different SOAs (−400 ms, A50V, PSS, V50A, 400 ms) as within-subject factors to test whether the behavioral results are different under different SOAs. The results showed that the main effect of SOAs was significant, [*F*_(4,95)_ = 68.31, *P* < 0.001, η*p^2^* = 0.782]. Further *post hoc t*-tests with Bonferroni correction revealed significant differences between the condition −400 ms vs. A50V SOA [*t*_(19)_ = −11.15, *P* = 8.83e-09], −400 ms vs. PSS SOA [*t*_(19)_ = −22.51, *P* = 0], −400 ms vs. V50A SOA [*t*_(19)_ = −8.23, *P* = 1.18e-06], −400 ms vs. 400 ms [*t*_(19)_ = −3.82, *P* = 0.012], A50V SOA vs. PSS [*t*_(19)_ = −9.47, *P* = 0], A50V SOA vs. 400 ms [*t*_(19)_ = 3.89, *P* = 0.01], PSS vs. V50A SOA [*t*_(19)_ = 6.64, *P* = 2.40e-05], PSS vs. 400 ms [*t*_(19)_ = 9.75, *P* = 0], V50A SOA vs. 400 ms [*t*_(19)_ = 5.16, *P* = 0.001]. There was no significant difference between the A50V and V50A SOAs [*t*_(19)_ = 0.01, *P* = 0.992]. We used a one-sample *t*-test to test whether the mean of the proportions of synchronous responses was about 50% at the A50V and V50A SOAs. The results indicated that there was no significant difference against 50% at the two SOAs [A50V: 0.546 ± 0.142, *t_*A*50*V*_*_(19)_ = 1.45, *P* = 0.163; V50A: 0.546 ± 0.223 (MEAN ± SD), *t_*V*50*A*_*_(19)_ = 0.915, *P* = 0.372]. These EEG-behavioral results suggest that the subjects’ responses under different SOAs are not random. The A50V and V50A SOA employed here effectively caused synchronous and asynchronous responses in about half of the trials.

### 3.2. EEG results

Given the presumed role of prestimulus phases of delta and theta frequency bands in shaping subsequent perceptual responses, we quantified the relationship between the delta, theta, and alpha oscillations phase and the perceptual responses in the A50V and V50A SOA with POS index, respectively. The POS of delta (1–3 Hz), theta (4–7 Hz), and alpha (8–13 Hz) bands were averaged over frequencies and submitted to a one-tailed dependent-samples *t*-test (empirical level POS vs. chance level POS) within −800 to 200 ms time range which also is used by one study (ten Oever and Sack, 2015) and 60 channels with spatio-temporal cluster correction using the Monte Carlo randomization method (1,000 repetitions) in the A50V SOA.

**FIGURE 4:**
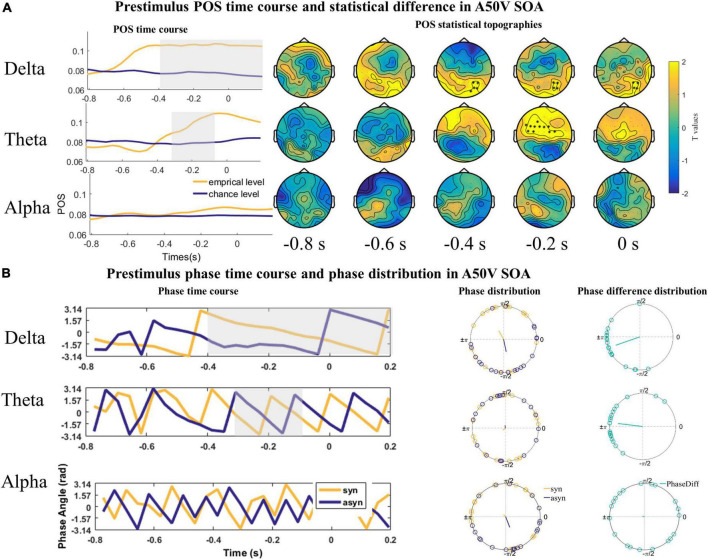
The statistical difference of phase opposition sum (POS) in the A50V stimulus onset asynchronous (SOA). **(A)** Left is the time course of empirical and chance level POS in the delta, theta, and alpha band respectively. Time 0 s indicates the onset of auditory signals. Two positive significant clusters were found in the delta and theta bands. Each topography shows the average t-map across a 200 ms temporal window starting at the indicated time point. **(B)** The time courses of phases extracted from and averaged over the cluster (delta and theta) or the whole channels (alpha) across subjects were shown on the left. The mean phases and phase differences are plotted for each participant in the delta, theta, and alpha band on the right. The orange, blue, and green lines indicate the mean resultant vector lengths (MRVLs). Gray-shaded areas indicate the timeframes with significant differences. Significant channels are highlighted with asterisks.

The result revealed two significant positive clusters. The first positive cluster was in a time window ranging from −400 to 200 ms in the delta band over right parieto-occipital sensors (cluster value = 399.94, *p* = 0.046; Figure 4A). The second positive cluster was in a time window ranging from −300 to −110 ms in the theta band over frontocentral sensors (cluster value = 386.75, *p* = 0.028; Figure 4A). No cluster was found in the alpha (8–13 Hz; Figure 4A) band.

The phases might encode temporal information rather than the excitability of the cerebral cortex. Thus, the phases under the same response across the subjects should be uniformly distributed. And the phase differences between the synchronous and asynchronous responses across subjects should be concentrated around π. To illustrate the nature of the phase effects underlying the significant POS effects, we extracted the phase angles for the single participant within the channel and time with the maximum *t* value of the cluster in the POS statistics and averaged over the frequency range in the delta (1–3 Hz) and theta (4–7 Hz) band for the Rayleigh and *V* tests. The Rayleigh test indicated that the mean phase over participants was inconsistent for both the delta and theta band (*Z*_delta_syn_ = 0.197, *P* = 0.825; *Z*_delta_azsyn_ = 0.153, *P* = 0.861; *Z*_theta_asyn_ = 2.922, *P* = 0.052, *Z*_theta_syn_ = 1.803, *P* = 0.166, Figure 4B). However, the *V* test showed that the phase difference between the synchronous and asynchronous responses in the two bands had a significant phase opposition and was concentrated around π [*V*_delta_ = 14.895, *P* < 0.001; *V*_theta_ = 14.557, *P* < 0.001, Figure 4B].

**FIGURE 5:**
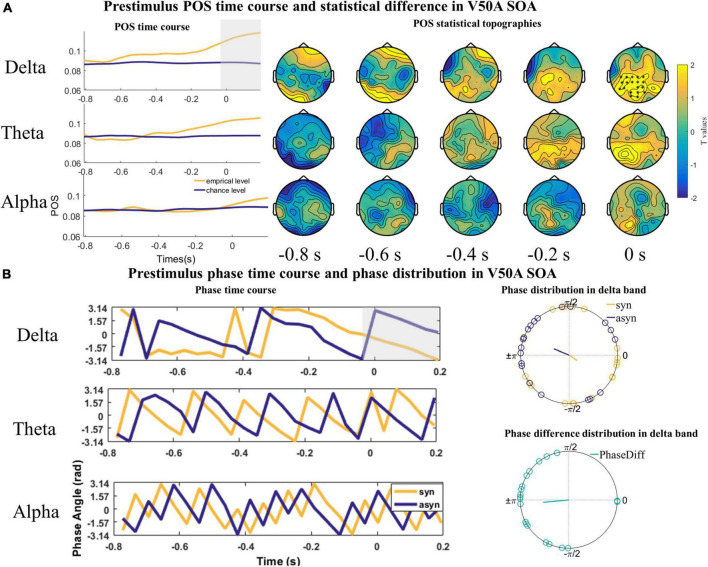
The statistical difference of phase opposition sum (POS) in the V50A stimulus onset asynchronous (SOA). **(A)** Left is the time course of empirical and chance level POS in the delta, theta, and alpha band respectively. Each topography shows the average t-map across a 200 ms temporal window starting at the indicated time point. Time 0 s indicates the onset of visual signals. **(B)** The time courses of phases extracted from and averaged over the cluster (delta) or the whole channels (theta and alpha) across subjects were shown on the left. The mean phases and phase differences are plotted for each participant in the delta band on the right. The orange, blue, and green lines indicate the mean resultant vector lengths (MRVLs). Gray-shaded areas indicate the timeframes with significant differences. Significant channels are highlighted with asterisks.

However, we only found one significant positive cluster in a time window ranging from −38 to 200 ms in the delta band over parieto-occipital and central sensors (cluster value = 366.46, *p* = 0.035; Figure 5A) in the V50A SOA. The Rayleigh test results at 0 s were not significant (*Z*_delta_syn_ = 1.115, *P* = 0.332; *Z*_delta_asyn_ = 1.738, *P* = 0.177, Figure 5B). However, the *V* test results indicated that the mean of the phase difference between synchronous and asynchronous responses was π [*V*_delta_ = 9.598, *P* < 0.001, Figure 5B]. No significant cluster was found in the theta (cluster value = 257.24, *p* = 0.076; Figure 5A) and alpha frequency band (cluster value = 20.962, *p* = 0.648; Figure 5A).

The results of the Rayleigh and *V* test in the A50V and V50A SOA indicate that individual participants have an optimal phase corresponding to synchronous and asynchronous response, but this exact phase in which participants make a synchronous or asynchronous response is not consistent among participants.

## 4. Discussion

In the current study, we propose a hypothesis that the ongoing oscillatory phase will shape the audiovisual speech temporal integration and segregation. We also suppose that the frequency of these oscillations changed as a function of the TBW size. We conducted a speech stimuli-based audiovisual simultaneity judgment (SJ) experiment recording EEG. The results revealed that the temporal perception of audiovisual speech mainly depends on the phase difference between the synchronous and asynchronous response in the delta-theta (1–7 Hz) band before the key stimulus onset. Moreover, the frequency range of these oscillations varies with the different audiovisual onset sequences (A50V and V50A SOA in this study) which correspond to different TBW sizes.

### 4.1. Relations between temporal perception and ongoing oscillations

Previous work has found that the phases of different ongoing oscillatory rhythms determine the temporal integration/segregation of visual stimuli according to the TBW size (Ronconi et al., 2017). Similar to the above result, we found a mapping between the phase of oscillatory activity at 1–7 Hz and the width of the TBW in the cross-modal audiovisual speech domain. Specifically, synchronous or asynchronous perceptual response in the A50V or V50A SOA depends on opposite prestimulus phases in the 1–7 Hz or 1–3 Hz frequency band respectively, whose periods are greater than the size of auditory or visual leading TBWs (198 or 273 ms). Combined with the alpha band phase modulation in beep-flash stimuli, our findings suggest that there might be also a precise mapping between oscillatory activity at a specific frequency and the temporal organization of sensory inputs into coherent percepts in the cross-modal domain. Furthermore, different oscillatory rhythms provide a hierarchical framework for the temporal organization corresponding to varying precisions. And our brain can flexibly use neural oscillations to ensure the continuity of time perception.

An important reason for the delta-theta band (1–7 Hz) effect on the temporal perception of audiovisual speech might be the neuronal entrainment effect (Lakatos et al., 2019). In natural speech, rhythmic alternation of syllables constitutes slow (∼1–8 Hz) temporal fluctuations of the acoustic speech signal. This speech rhythm is tracked by phase-locking endogenous oscillations, known as neural entrainment, which has been proven to play a crucial role in speech perception, especially in temporal perception. For example, Several previous studies have shown that audiovisual speech dynamic tracking (Luo et al., 2010) or short acoustic target detection (Ng et al., 2012) were modulated by the phase of the low-frequency delta-theta band. Furthermore, the audiovisual temporal integration effect in natural speech processing occurs in the delta-theta band (1–7 Hz), i.e., the timescale of the syllable (Crosse et al., 2015, 2016).

Notably, given the flexibility and adaptability of our brains, we believe that the brain could use different rhythms to parse temporal perception for speech stimuli, which depends on the stimulus and context. For example, the recognition of spoken languages at different speaking rates requires us to adapt flexibly, and many studies have shown that it will affect the temporal perception of speech (Bosker and Ghitza, 2018; Kösem et al., 2018). The syllables used in this study are slightly slower than the normal pronunciation rate, and a previous study has shown that the pronunciation speed of words affects the width of TBW (Bhat et al., 2015). So, the speech rates might be a vital factor for the phase modulation of different oscillation rhythms in audiovisual speech stimuli and should be studied further. In addition, it has been demonstrated that although syllable recognition of auditory speech is modulated by the phase of ongoing 6.25 Hz, it also depends on the underlying oscillatory phase induced by the entrained 10 Hz neural oscillations (ten Oever and Sack, 2015).

### 4.2. The apparent asymmetry of temporal perception

Our results indicated that the temporal perception in A50V and V50A conditions is modulated by the phase of different frequency bands, which might be due to the different widths of auditory and visual leading TBW. According to the behavior results, auditory leading TBW is about 198 ms, while visual leading TBW is about 273 ms. It means A50V may be affected by a higher frequency (∼5 Hz) with a shorter oscillation period and V50A by a slightly lower frequency (∼3.5 Hz) with a more extended oscillation period. The exciting result is that the delta phase also affects the response in the A50V condition, and the topography of the delta phase effect is in the right parieto-occipital region. This is also reasonable since it is theoretically possible to modulate the perceptual response as long as the period of neural oscillations is longer than the width of TBW. It is well-known that the response to visual stimuli mainly occurred in the parieto-occipital region. In contrast, the response to auditory stimuli appeared in the temporal and middle frontal areas. A reasonable guess is that the phase modulation in the A50V condition is affected by both visual and auditory brain regions. In contrast, the phase modulation under the V50A condition is more affected by the visual area. Since the scalp EEG does not have a high spatial resolution, future studies need to use a high spatial resolution technique to explore the role of different brain regions.

### 4.3. The role of EEG phases: Excitability or phase coding

The prevailing view holds the opinion that EEG phases represent the excitatory states of the brain (Ronconi et al., 2017). Our study, however, shows different results. If the phase represents the cortical excitability, then the phase under the same response across subjects will have an aggregation effect. However, the results of the Rayleigh test indicated that the phase does not concentrate in a single phase over participants but presents a uniform distribution. Moreover, the results of the *V* test show that the phase difference of synchronous and asynchronous responses concentrates around π, indicating that different responses of the same subject are modulated by opposite phases. Thus, we support the opinion that the phases encoding the time information of audiovisual onset difference for speech vary across subjects, which is similar to the previous study (ten Oever and Sack, 2015).

## 5. Conclusion

Overall, we investigate the role of the oscillatory phase for simultaneity judgments on audiovisual speech stimuli. That is, visual and acoustic speech cues are delayed relative to each other and participants indicate whether they perceived the audiovisual asynchronous speech stimuli as synchronous. Our study demonstrates that the brain could use the phases of different oscillatory rhythms flexibly to organize temporal perception with varying precision for speech stimuli. The role of the oscillatory phase is to encode temporal information which varies across subjects.

## Data availability statement

The raw data supporting the conclusions of this article will be made available by the authors, without undue reservation.

## Ethics statement

The studies involving human participants were reviewed and approved by Ethics Committee of Tianjin University. The patients/participants provided their written informed consent to participate in this study. Written informed consent was obtained from the individual(s) for the publication of any potentially identifiable images or data included in this article.

## Author contributions

DM and XA contributed to the conception and design of the study. XA revised the manuscript and supervised the entire study. ZJ and LW conducted the experiment and performed data analysis. ZJ drafted the manuscript. EY, SL, and YY revised the manuscript. All authors contributed to the article and approved the submitted version.
